# A Minimum Temporal Window for Direction Detection of Frequency-Modulated Sweeps: A Magnetoencephalography Study

**DOI:** 10.3389/fpsyg.2020.00389

**Published:** 2020-03-10

**Authors:** Shu-Jen Kung, Denise H. Wu, Chun-Hsien Hsu, I-Hui Hsieh

**Affiliations:** ^1^Institute of Cognitive Neuroscience, National Central University, Taoyuan City, Taiwan; ^2^Institute of Linguistics, Academia Sinica, Taipei, Taiwan

**Keywords:** frequency sweep, mismatch negativity, magnetoencephalography, speech encoding, temporal window

## Abstract

The ability to rapidly encode the direction of frequency contour contained in frequency-modulated (FM) sweeps is essential for speech processing, music appreciation, and conspecific communications. Psychophysical evidence points to a common temporal window threshold for human listeners in processing rapid changes in frequency glides. No neural evidence has been provided for the existence of a cortical temporal window threshold underlying the encoding of rapid transitions in frequency glides. The present magnetoencephalography study used the cortical mismatch negativity activity (MMNm) to investigate the minimum temporal window required for detecting different magnitudes of directional changes in frequency-modulated sweeps. A deviant oddball paradigm was used in which directional upward or downward frequency sweep serves as the standard and the same type of sweep with the opposite direction serves as its deviant. Stimuli consisted of unidirectional linear frequency-sweep complexes that swept across speech-relevant frequency bands in durations of 10, 20, 40, 80, 160, and 320 ms (with corresponding rates of 50, 25, 12.5, 6.2, 3.1, 1.5 oct/s). The data revealed significant magnetic mismatch field responses across all sweep durations, with slower-rate sweeps eliciting larger MMNm responses. A greater temporally related enhancement in MMNm response was obtained for rising but not falling frequency sweep contours. A hemispheric asymmetry in the MMNm response pattern was observed corresponding to the directionality of frequency sweeps. Contrary to psychophysical findings, we report a temporal window as short as 10 ms sufficient to elicit a robust MMNm response to a directional change in speech-relevant frequency contours. The results suggest that auditory cortex requires extremely brief temporal window to implicitly differentiate a dynamic change in frequency of linguistically relevant pitch contours. That the brain is extremely sensitive to fine spectral changes contained in speech-relevant glides provides cortical evidence for the ecological importance of FM sweeps in speech processing.

## Introduction

Frequency modulation (FM) is a fundamental acoustic component of all complex communication signals in speech, music, and conspecific vocalization. Frequency sweeps or glides are an important class of FM signals which provide informative cues for phonemic identification and lexical distinction in tonal languages. Manipulation of the frequency-modulated features of speech affects a range of perceptual experience such as meanings and emotions conveyed in language. Previous studies have suggested that perceptual processing of frequency sweeps is modulated by language experience, associated with musical abilities (d’Alessandro et al., 1998; Gordon and Poeppel, 2002; Luo et al., 2007; Pfordresher and Brown, 2009; Hove et al., 2010), auditory scene analysis (Carlyon, 1994), and can be observed in the spectrogram of crying intonations from a newborn (Mampe et al., 2009). Deficits in processing FM sounds have been shown to be associated with the development of reading ability (Boets et al., 2011; Wang et al., 2019), language-based learning impairment (Tallal, 1980; Stefanatos et al., 1989; Neville et al., 1993; Talcott et al., 2000; Stoodley et al., 2006), and phonological skill acquisition (Talcott et al., 2000). They also provide important applications for cochlear implant users (Chen and Zeng, 2004).

The acoustic information contained in FM sweeps includes frequency directionality, frequency bandwidth, and temporal scales, which play significant roles in language processing. In tonal languages, such as Mandarin Chinese and Thai, variations in frequency sweep contours over relatively long time scales of 200–300 ms provide dominant cues for tone recognition and for determining different lexical meanings in syllables (Yip, 2002; Liu et al., 2007). For example, the segmental sequence (ma) conveys four distinct meanings in Mandarin Chinese when spoken with combinations of high level, high rising, or low dipping contours. Directional FM sweeps also provide important cues for English (a non-tonal language) listeners in discriminating phonemes (i.e., /ba/from/da/) over short time scales of 20–30 ms (Miller and Liberman, 1979; Kraus et al., 1994; Poeppel, 2003). Despite reported threshold differences between tone-language and non-tone language speakers in identifying the direction of FM sweeps, a common temporal integration window of 20 ms has been suggested by several psychophysical studies for constructing an elementary auditory percept for perceptual differentiation (Poeppel, 2003; Luo et al., 2007). These studies indicate that the time scales over which a change in the directionality of FM sweeps can be encoded, both perceptually and at the cortical level, plays an essential role in the early step in the processing of speech streams (Joanisse and Gati, 2003; Hickok and Poeppel, 2004; Joanisse and DeSouza, 2014).

Human brain imaging studies investigating the neural basis underlying the encoding FM-sweep features have mostly focused on either the modulation rate or directional features with inconsistent results. Previous fMRI studies using multivariate pattern classification analysis identified two brain regions, the right primary auditory cortex and the left superior temporal gyrus, as selective sites for FM direction processing using slower-rate FM sweeps (i.e., 0.83 and 3.3 oct/s) (Hsieh et al., 2012). Joanisse and DeSouza (2014) used iterated rippled noise stimuli modulated by faster FM rates (20 oct/s and 10 oct/s) and reported similar activation in the bilateral auditory cortex that are selective for FM sweep direction. However, they reported no evidence for feature-specific encoding of the rate of frequency modulation at this time scale of FM sweeps. FM sweep processing has also been investigated using the deviant oddball paradigm, in which an evoked auditory potential peaked around 100–250 ms after the onset of a stimulus change was assumed to index cortical detection of a change in the neural memory trace for the feature of interest (i.e., pitch, intensity) (Näätänen et al., 1993; Ritter et al., 1995; Picton et al., 2000; Zuijen et al., 2006). Previous magnetoencephalography (MEG) studies have reported on the magnetic counter part of MMN (MMNm) in the right auditory cortex elicited by a directional change for 100-ms FM sweeps spanning one octave in range, centered at different frequencies. An enhancement in MMNm response has also been noted when successive FM sweeps were presented in identical directions (Heinemann et al., 2010; Altmann et al., 2011). However, Altmann et al. (2011) showed that a deviation present in a repeated series of FM sweeps did not invoke significant directional change-related enhancements. Higher mismatch negativity amplitude has also been reported in response to tone deviants with ascending than descending contours within a melodic sequence but only when the tones occurred in unequal probabilities (Paavilainen et al., 1999; Gumenyuk et al., 2003; Ruusuvirta and Astikainen, 2012). Interestingly, even newborn infants demonstrate the ability to discriminate ascending to descending frequency tone pairs in a melodic sequence (Saarinen et al., 1992; Carral et al., 2005). Several studies using discrete tones at different fixed frequencies in a sequence have also reported increments in frequency produced higher MMN amplitudes compared to frequency decrements (Jacobsen and Schröger, 2001; Peter et al., 2010; Karanasiou et al., 2011). Additionally, Okamoto and Kakigi (2017) reported that the evoked auditory N1m response was smaller for upward sweeping FM sounds modulated at high FM rates, with the response most pronounced in the right hemisphere. Taken together, human neuroimaging results point to directional selective sites for FM sweep processing with inconsistent results on the processing of FM-sweep rates. Findings from MMN/MMNm studies suggest that the auditory cortex is able to pre-attentively extract abstract relationships between transitions of frequency-sweep directions (Sams and Näätänen, 1991; Pardo and Sams, 1993), but the minimum temporal window required for encoding a change in frequency sweep contours has not be clarified.

Overall, previous studies employing the MMNm paradigm to examine the brain’s pre-attentive processing of FM features at an automatic preconscious encoding level have mostly focused on detecting changes in the directionality of frequency contours. In general, the frequency sweep durations examined in these MMNm studies were relatively long (i.e., slow rates), ranging from approximately 100 – 250 ms (Pardo and Sams, 1993) to 1000 ms (Okamoto and Kakigi, 2015). It has been suggested that the duration limit or “temporal window” of the frequency contour, at which the auditory cortex is able to reliably detect direction, can critically affect how speech sounds are perceived (Hickok and Poeppel, 2000). Psychophysical findings on FM-sweep detection have suggested a temporal integration window of 20 ms for constructing an elementary auditory percept for perceptual differentiation (Luo et al., 2007). However, it is unclear whether a similar temporal window limit exists at the cortical level for the detection of a spectral change in frequency sweep contours.

The aim of the present study was to investigate the minimum sweep duration (rate) required for the auditory cortex to automatically encode a change of spectral direction in frequency sweep contours. The evoked magnetic field responses to unexpected variations of the FM direction in an oddball paradigm was recorded for sweep durations ranging from 10 ms to 320 ms (corresponding to rates of 1.5–50 oct/s) in speech-relevant frequency bands (Luo et al., 2007). To prevent the use of potential spectral edge pitch cues in detecting frequency direction, the frequency sweeps were bounded by two pure steady-state tones (Hsieh et al., 2012). Based on previous findings, it is expected that the extent of the directional change in FM sweep contours would correspond to a cortical frequency-change detection indexed by the strength of the MMNm response. More specifically, given that psychophysical studies have reported a common temporal threshold of 20 ms in detecting FM sweep direction, it is reasonable to speculate that a neural change detection window should exist corresponding to frequency sweeps of at least the same duration/rate. We report a surprisingly short frequency sweep duration of 10 ms sufficient to elicit a robust MMNm response. This result suggests that the brain requires extremely short temporal window to implicitly differentiate fine acoustic spectral changes at the pre-attentive cortical level.

## Materials and Methods

### Participants

Fourteen native Mandarin Chinese speakers (8 females, mean age = 22.4, range 19 –27 years) with normal hearing (self-assessed) participated in the experiment. All participants were right-handed and reported no history of neurological or psychiatric disease. Participants were fully informed about the study and gave written informed consent for their participation. All procedures were approved and conducted in accordance with the Research Ethics Committee at National Taiwan University in Taiwan and the Human Subject Research Ethics Committee/Institutional Review Board of Academia Sinica, Taiwan.

### Stimuli

Stimuli were generated digitally using MATLAB software (Version 2015b; Mathworks Inc., Natick, MA, United States) on an ASUS Vento PC and presented at a rate of 44.1 kHz using 16-bit digital-to-analog converters (Creative Sound Blaster X-Fi Titanium). The stimuli consisted of unidirectional linear FM sweep complexes bounded by two steady-state pure tones. FM sweep was generated using the following equation:

$$X\,\left(t\right)=\sin\left(2\,\pi \,f_{s}\,\left(t\right)+\pi \,\frac{f_{e}-f_{s}}{T_{s}}\,{\left(t\right)}^{2}\right)$$

where *f*_*s*_ and *f*_*e*_ represent the starting and ending sweep frequencies in hertz, and *T*_*s*_ and *t* are the stimulus duration and time in seconds, respectively.

**FIGURE 1:**
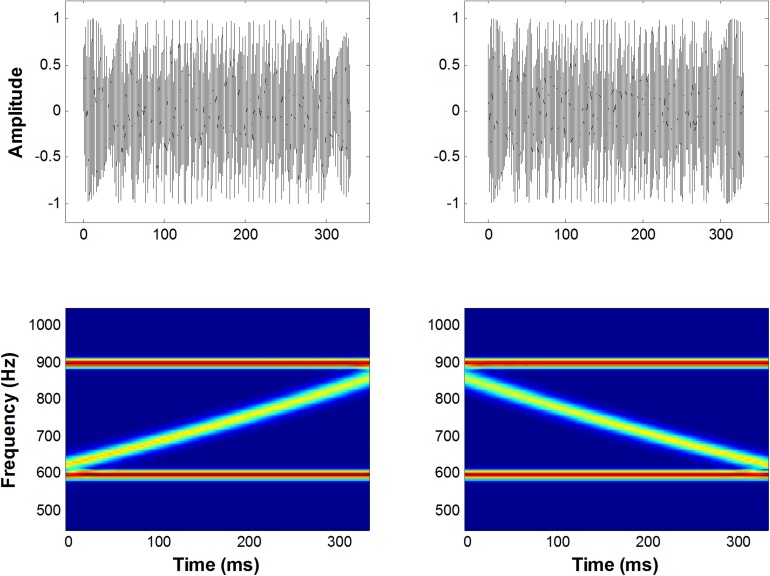
A schematic representation of the up and down FM sweep complex. The left panel shows an amplitude spectrum **(top)** and a spectrogram **(bottom)** for upward FM sweeps in a frequency range of 600–900 Hz, bounded by two fixed-frequency pure tones. The right panel shows the same illustration for downward FM sweeps.

A diagram of the FM sweep stimuli and spectrogram is shown in Figure 1. The auditory stimuli consisted of 12 conditions (6 × 2 design), six stimulus durations (*T_*s*_* = 10, 20, 40, 80, 160, and 320 ms), and two sweep directions (up or down in frequency). Both the rising and falling FM sweeps spanned a frequency range of 600–900 Hz at six different rates. FM rate is parameterized by varying the duration of the signal while keeping the bandwidth of all FM stimuli constant at half an octave (i.e., 600–1200 Hz = 1 octave). The following FM durations (with corresponding FM-sweep rates) were examined: 10 ms (50 oct/s), 20 ms (25 oct/s), 40 ms (12.5 oct/s), 80 ms (6.2 oct/s), 160 ms (3.1 oct/s), and 320 ms (1.5 oct/s). All stimuli were superimposed with linear rise and decay ramps of 2 ms to minimize spectral splatter and were presented diotically via MEG-compatible headphones. The extent and rate of these sweeps were in the general range of those observed during formant transitions and frequency glides in speech (Kewley-Port, 1982), as well as the range used in most psychophysical and neuroimaging studies of directional FM sweeps (Poeppel et al., 2004; Brechmann and Scheich, 2005; Luo et al., 2007; Hsieh and Saberi, 2010). Two boundary tones were also added to these FM sweeps to eliminate cues related to a shift in the centroid of the spectral energy between the start and end points of the stimuli. The low boundary component was a pure tone at the lowest FM component (i.e., 600 Hz), while the high boundary pure tone started at the highest frequency component (i.e., 900 Hz). Both tones remained at a constant frequency for the duration of the stimulus. The initial and final frequencies of the standard and deviant sweeps were equivalent for each condition. The amplitudes of the two boundary tones were attenuated by 6 dB (a half amplitude) using a logarithmic ramp as the frequency of the FM sweep stimulus approached that of the boundary tone (Hsieh et al., 2012). The sweep stimulus level was calibrated to 70 dB SPL using a 6-cc coupler, 0.5-in. microphone (Brüel & Kjær, Model 4189), and a precision sound analyzer (Brüel & Kjær, Model 2260).

### Procedure

Each participant was tested individually in a silent, magnetically shielded room located at Academia Sinica in Taiwan. During the experiment, the neural response of each participant was recorded continuously using the MEG system (Kanazawa Institute of Technology, Kanazawa, Japan) as the individual viewed a silent movie. Auditory stimuli were delivered through inserted earpieces (ER30, Etymotic Research Inc.) connected to plastic tubes and presented at an intensity level of 70 dB SPL. The stimuli consisted of a sequence of repetitively presented standard tones interspersed by infrequent deviant tones, with an inter-stimulus interval of 700 ms. These stimuli were pseudo-randomized within a block and included constraints requiring at least two standard tones between two deviant tones. Each FM-sweep block started with 20 presentations of the standard stimuli, which were followed by 320 trials with 60 presentations of deviant sounds (i.e., an 18.75% deviant probability) and 260 standard sounds (81.25% standard probability). Each participant received a total of 60 presentations of deviant sounds per each of the 12 FM-sweep blocks. McGee et al. (2001) has suggested that at best only 100–125 deviant stimuli should be presented under approximately 10–12 min before habituation starts to degrade the MMN response. In order to minimize the potential MMN habituation effects suggested to occur if sessions are too long (McGee et al., 2001), we have minimized the stimuli presentation in short sequences with block durations ranging between 3 min 47 s (10-ms sweeps) and 5 min 26 s (320 ms sweeps).

Six different sweep durations (10, 20, 40, 80, 160, and 320 ms) and thus six sweep rates were tested in separate blocks. The direction of the FM sweeps was the only difference between the deviant and standard stimuli in each block. For example, in the 10 ms UP block (i.e., the UP10 condition), the deviant stimuli consisted of 10 ms upward FM sweeps while the standard stimuli included downward FM sweeps of 10 ms duration. In contrast, for the DOWN10 condition, the 10 ms downward FM sweeps served as the deviant stimuli in the context of 10 ms upward FM sweeps serving as the standard stimuli. Therefore, the experiment included a total of 12 blocks (6 duration × 2 direction). The presentation order of these blocks was randomized for each participant.

Following the MEG recording, participants then completed the FM sweep behavioral task. We chose to perform the behavioral task *after* the completion of the EEG recordings to prevent any possible bias towards sweep direction caused by attentively judging the FM tone complex. The behavioral task consisted of a FM direction identification task in a single interval, two alternative forced choice (2AFC) paradigm. The same FM stimulus conditions (6 durations) used in the MEG study were tested, except that the longest duration sweep was not tested due to pilot study showing that performance was at ceiling when sweep duration was above 40 ms. There were 100 trials in each of the six FM sweep conditions tested, giving a total of 600 trials for each participant. The experiment was run in a random block design in which the sweep duration served as separate blocks. In each trial, subjects had to judge the direction of the sweep as UP or DOWN in frequency by pressing one of two labeled keys. The inter trial interval was 600 ms. After half of the session, key reversal was used to eliminate key press bias. No feedback was given to the participant. All stimuli were presented binaurally through Sennheiser headphones (HD 380 Pro) at 70 dBA.

### MEG Recording and Data Analysis

The evoked magnetic fields were continuously recorded throughout the task using a whole-head 160-channel magnetometer system (Kanazawa Institute of Technology, Kanazawa, Japan) in a magnetically shielded chamber. A band-pass filter (0.03–100 Hz with a notch at 60 Hz) with a sampling frequency of 1 kHz was applied during the recording process. Raw data were noise-reduced using a recording of room noise in order to minimize environmental contamination. The magnetic field response of the brain was then off-line filtered with a band-pass filter of 1–30 Hz. The time period ranging from a 100 ms pre-stimulus to a 500 ms post-stimulus was used as an epoch, with the pre-stimulus period used as baseline correction. Epochs with field-gradient variations exceeding ±3 pT/cm in any magnetometer channel were excluded from subsequent analyses. On average, less than 15% of the total number of epochs were rejected and the remaining ∼280 artifact-free epochs for each condition were taken into averaging. After the artifact rejection step, any remaining epochs from the deviant and standard stimuli were averaged and low-pass filtered at 40 Hz separately for each participant.

### Analyzing MMNm Activity in MEG Waves

**FIGURE 2:**
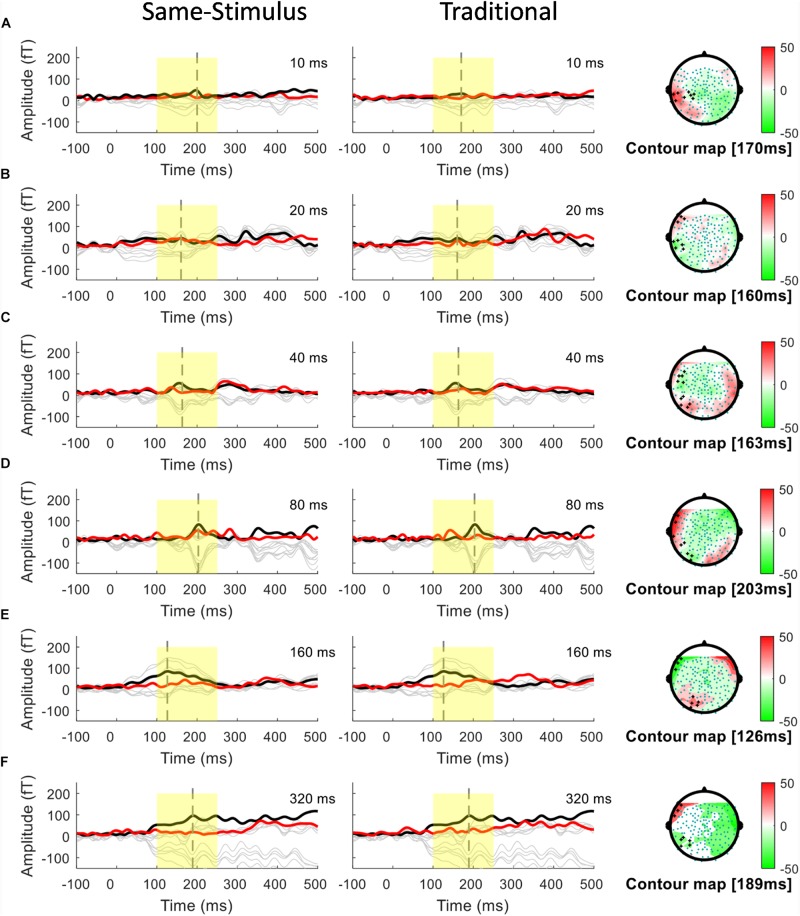
Overlays of average standard (red line) and deviant (black line) magnetic field waveforms from a representative participant for each upward FM-sweep duration condition in the left hemisphere. The red lines are the RMS of the standard waveforms that were measured from the 10 selected sensors that showed the field patterns of MMNm. The topographical maps (right) show the differential activities of the mismatched waveforms corresponding to each sweep condition. MMNm response is elicited similarly using the same-stimulus (left) and traditional (middle) methods. Standard and deviant waveforms elicited by **(A)** 10-ms sweep **(B)** 20-ms sweep **(C)** 40-ms sweep **(D)** 80-ms sweep **(E)** 160-ms sweep **(F)** 320-ms sweep.

**FIGURE 3:**
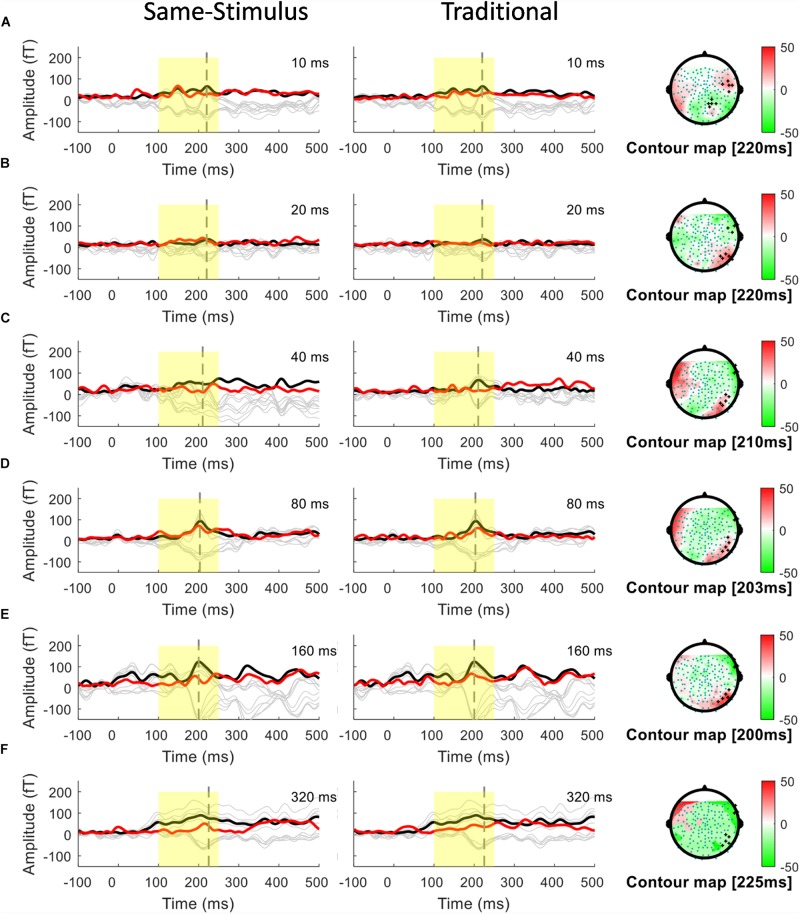
Overlays of average standard (red line) and deviant (black line) magnetic field waveforms from a representative participant for each upward FM-sweep duration condition in the right hemisphere. The red lines are the RMS of the standard waveforms that were measured from the 10 selected sensors that showed the field patterns of MMNm. The topographical maps (right) show the differential activities of the mismatched waveforms corresponding to each sweep condition. MMNm response is elicited similarly using the same-stimulus (left) and traditional (middle) methods. Standard and deviant waveforms elicited by **(A)** 10-ms sweep **(B)** 20-ms sweep **(C)** 40-ms sweep **(D)** 80-ms sweep **(E)** 160-ms sweep **(F)** 320-ms sweep.

A conventional approach for analyzing MMN/MMNm response was used by subtracting the activities to the standard from those to the deviant to determine the difference waves. We selected 10 sensors in the left and right hemispheres to do the following analyses. Traditional mismatch waveforms were obtained by subtracting the magnetic field response to the standard up sweep stimulus from the response to the deviant down sweep stimulus in the same block, respectively. Ten channels with maximum magnetic amplitude (5 channels in sink and 5 in source) over each hemisphere, located above the temporal lobes, were selected for individual participants based on the grand mean of the MMNm response (averaged over all 12 conditions). The MMNm response with the magnetic pattern of the polarity inversion across the lateral sensors during the time window from 150 to 300 ms was selected (Phillips et al., 2000). For each participant and each condition, different sets of sensors were selected over the two hemispheres. The peak latency of the MMNm component for each condition was determined for individual participants. The MMNm latency was defined as the time point with the largest Root-Mean Square (RMS) waveform amplitude from the 10 selected source channels within a temporal window of 150 – 300 ms post stimulus-onset. The MMNm amplitude was determined for each condition and each participant using the mean of the RMS values across a ±20 ms time window centered at the MMNm peak latency in each hemisphere. Figures 2, 3 show overlays of the average standard (red line) and deviant (black line) waveforms from a representative participant for each upward sweep duration condition in the left (Figure 2 middle panel) and right (Figure 3 middle panel) hemisphere, respectively. The red lines represent the RMS of the standard waveforms that were measured from the 10 selected sensors that showed the field patterns of MMNm. The topographical maps show the differential activities of the mismatched waveforms corresponding to each FM sweep duration condition.

### Analyzing Same-Stimulus MMNm Activity in MEG Waves

To examine the potential confound to MMNm response affected by acoustic feature difference between the standard and deviant stimuli, several groups have suggested using the same-stimulus method, which computes the ERP difference between the same stimulus presented as the standard in one block and as deviant stimulus in another block (Jacobsen and Schröger, 2003; Peter et al., 2010). This method may help reveal whether the MMNm response elicited by FM sweeps is affected by some inherent acoustic differences between the upward and downward FM sweeps. For each FM sweep duration, the same-stimulus MMNm was computed by subtracting the ERP to the identical sweep presented as a standard in one block (i.e., DOWN condition) and a deviant in another block (i.e., UP condition). For example, the same-stimulus MMNm response for the 10 ms up sweep condition was calculated by subtracting the ERP to the 10-ms up sweep in the DOWN10 (i.e., up as standard) block from the ERP to the same 10-ms up sweep in the UP10 (i.e., up as deviant) block. The resulting same-stimulus MMNm are compared with the MMNm computed using the traditional method to see if there is any difference in MMNm amplitude. Overlays of the average standard (red line) and deviant (black line) waveforms from a representative participant for each upward sweep duration condition calculated using the same-stimulus method is shown in the left panels in Figures 2, 3 for the left and right hemispheres, respectively.

## Results

### MMNm Response for FM Sweep Conditions

**FIGURE 4:**
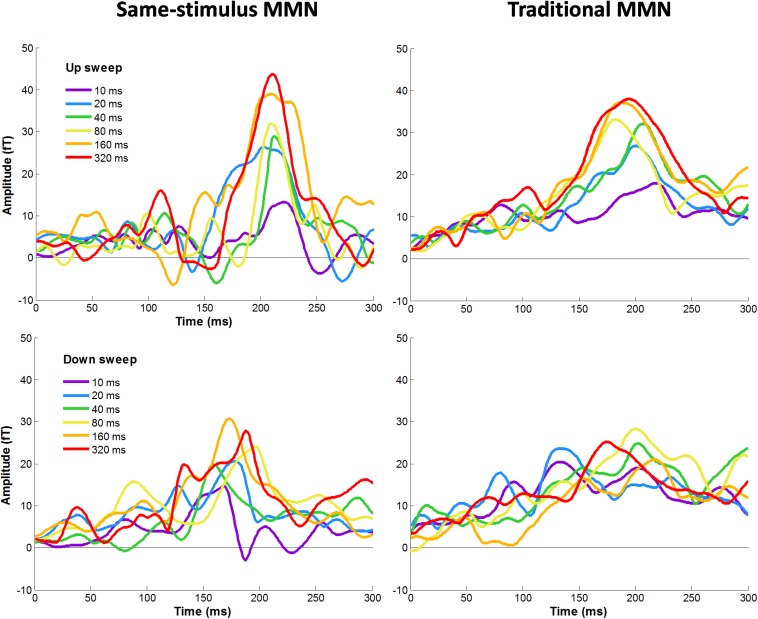
Grand-averaged MMNm responses for each upward **(top panel)** and downward **(bottom panel)** FM sweeps as a function of sweep duration. Left and right panels show MMNm waveforms calculated using same-stimulus and traditional methods, respectively. Different color lines represent different sweep duration condition. Responses were averaged across hemispheres.

Figure 4 shows average difference waves for the mean RMS calculated between standard and deviant stimuli for each condition calculated using same-stimulus and traditional methods. We first evaluated the effects of MMNm, recorded using sensors covering the temporal areas, by comparing difference waves from the mean RMS with zero values for each of the sweep condition. The amplitude of the MMNm response computed using the traditional method reached statistical significance for each of the 12 sweep conditions (6 durations × 2 directions), all *ts* (13) > 3.32, *ps* < 0.005. The amplitude of the MMNm response calculated using the same-stimulus method also reached statistical significance for each of the 12 sweep conditions (6 durations × 2 directions), all *ts* (13) > 6.22, *ps* < 0.005.

### Effects of Sweep Feature on MMNm Peak Latency

**FIGURE 5:**
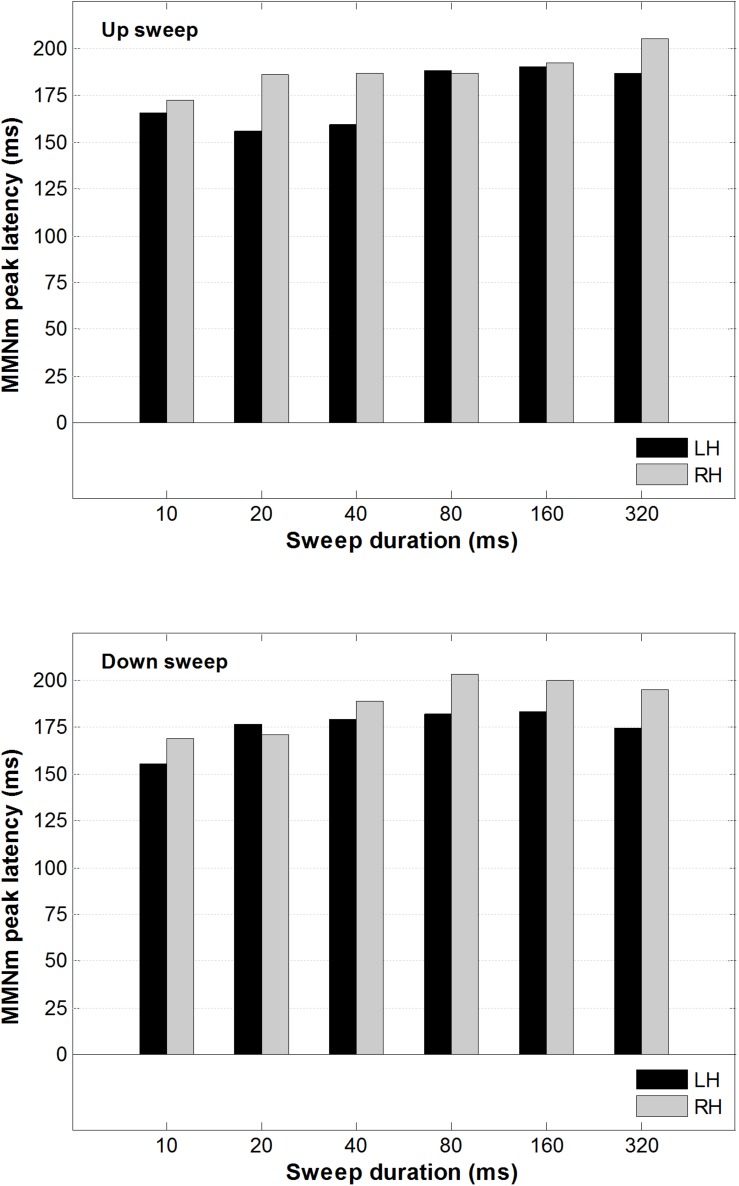
Average MMNm peak latency for up and downward FM sweeps as a function of sweep duration. Black and gray bars represent peak latency for left and right hemisphere, respectively. No hemispheric difference was observed in the MMNm latency for both upward and downward FM sweeps.

A 2 × 2 × 6 repeated-measure analysis of variance (ANOVA) was conducted on the peak latency of the MMNm, with factors including hemisphere (left vs. right), direction (up vs. down), and duration (10, 20, 40, 80, 160, and 320 ms). This was done to determine whether the peak latency of the MMNm was modulated by FM sweep direction or rate. As illustrated in Figure 5, the peak latency of the MMNm occurred between 150 and 200 ms after stimulus onset for most of the sweep conditions. There was no significant main effect of hemisphere, *F*(1,13) < 1, sweep direction *F*(1,13) = 2.20, *p* = 0.162, or duration *F*(5,65) = 1.214, *p* = 0.31 on MMNm peak latency.

### Effects of Sweep Feature on MMNm Peak Amplitude

**FIGURE 6:**
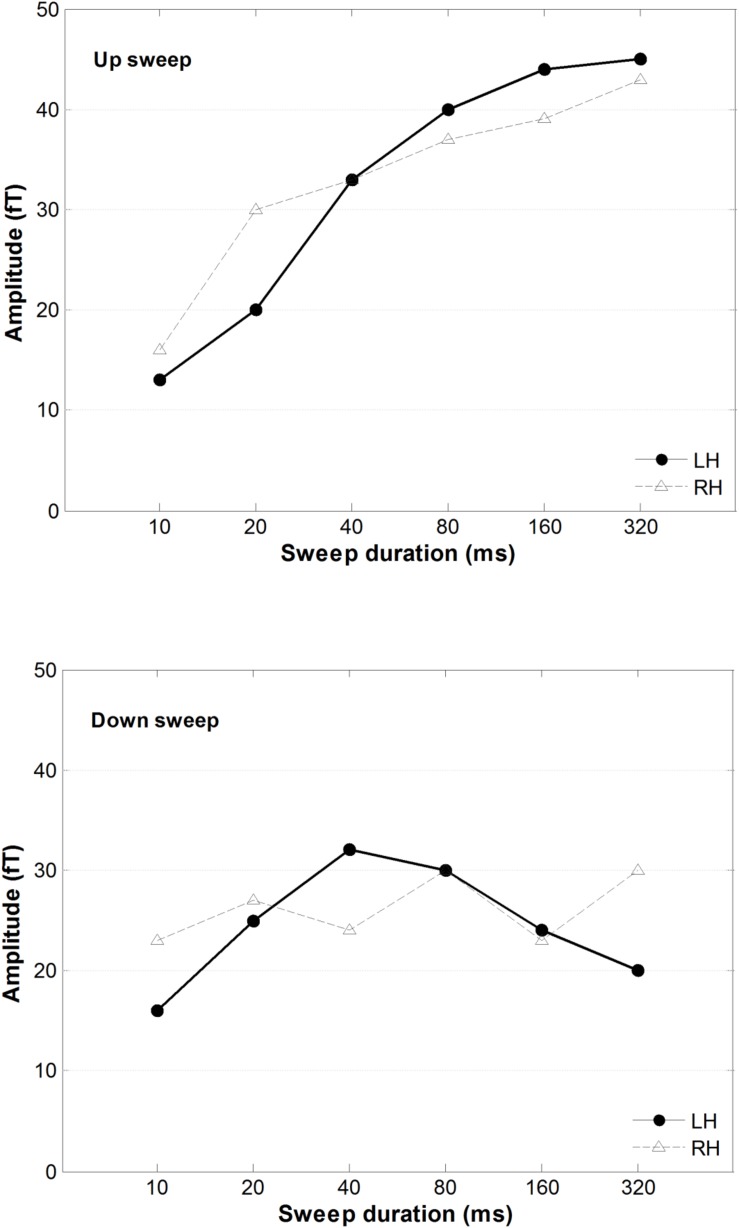
The averaged MMNm peak amplitudes for upward **(top)** and downward **(bottom)** FM sweeps as a function of sweep duration. Solid and dashed lines indicate response amplitude in the right and left hemisphere, respectively.

A 2 × 2 × 6 repeated-measure ANOVA was conducted on the mean of the RMS of the magnetic power, using hemisphere, direction, and duration as variance factors. As illustrated in Figure 6, upward frequency sweeps elicited a larger MMNm response than downward sweeps, *F*(1,13) = 39.33, *p* < 0.001. There was a significant main effect of sweep duration on MMNm power, *F*(5,65) = 6.46, *p* < 0.001. The interaction between sweep direction and duration on MMNm amplitude was statistically significant, *F*(5,65) = 3.89, *p* = 0.004. A *post hoc* comparison using Tukey’s HSD test revealed that the amplitude of the MMNm increased in upward sweeps as the sweep duration increased, but this trend was not observed in downward sweeps. Specifically, the MMNm amplitude was smaller for the 10 ms upward sweep compared to all other sweep duration conditions from DOWN40 to DOWN320 ms (all *ps* < 0.05). The MMNm amplitude of the 20 ms upward sweep was smaller than in the DOWN160 and DOWN320 ms conditions (both *ps* < 0.05). However, the MMNm amplitude elicited by the down sweeps did not show any difference with respect to any other downward sweep duration.

### Effects of Sweep Feature on MMNm Peak Amplitude: Comparison of Same-Stimulus and Traditional Methods

Figure 4 illustrates the average MMNm waveforms elicited by upward and downward sweeps for each sweep duration calculated using the same-stimulus (left panels) and traditional (right panels) methods, respectively. A three-way repeated measure ANOVA was performed with the factors direction (up vs. down), duration (10, 20, 40, 80, 160, and 320 ms), and method of calculation (same-stimulus vs. traditional) on MMNm peak amplitude. Left and right-hemisphere responses are combined for this analysis. The ANOVA revealed a significant main effect of direction on the peak amplitude of the MMNm response. Specifically, upward FM sweeps elicited larger MMNm response than downward sweeps, *F*(1,25) = 10.51, *p* = 0.003. There was a significant effect of duration on mean MMNm amplitude, *F*(5,125) = 8.477, *p* < 0.001. The effect of method of calculation on MMNm amplitude was not significant, *F*(1,25) = 0.022, *p* = 0.883. The interaction between sweep direction and duration on MMNm peak amplitude was not significant, *F*(5,125) = 0.563, *p* = 0.729. There was no significant interaction between calculation method and sweep direction or duration on MMNm amplitude, both *Fs* < 1.

### Comparison of Same-Stimulus and Traditional MMNm Response to 10-ms FM Sweeps

**FIGURE 7:**
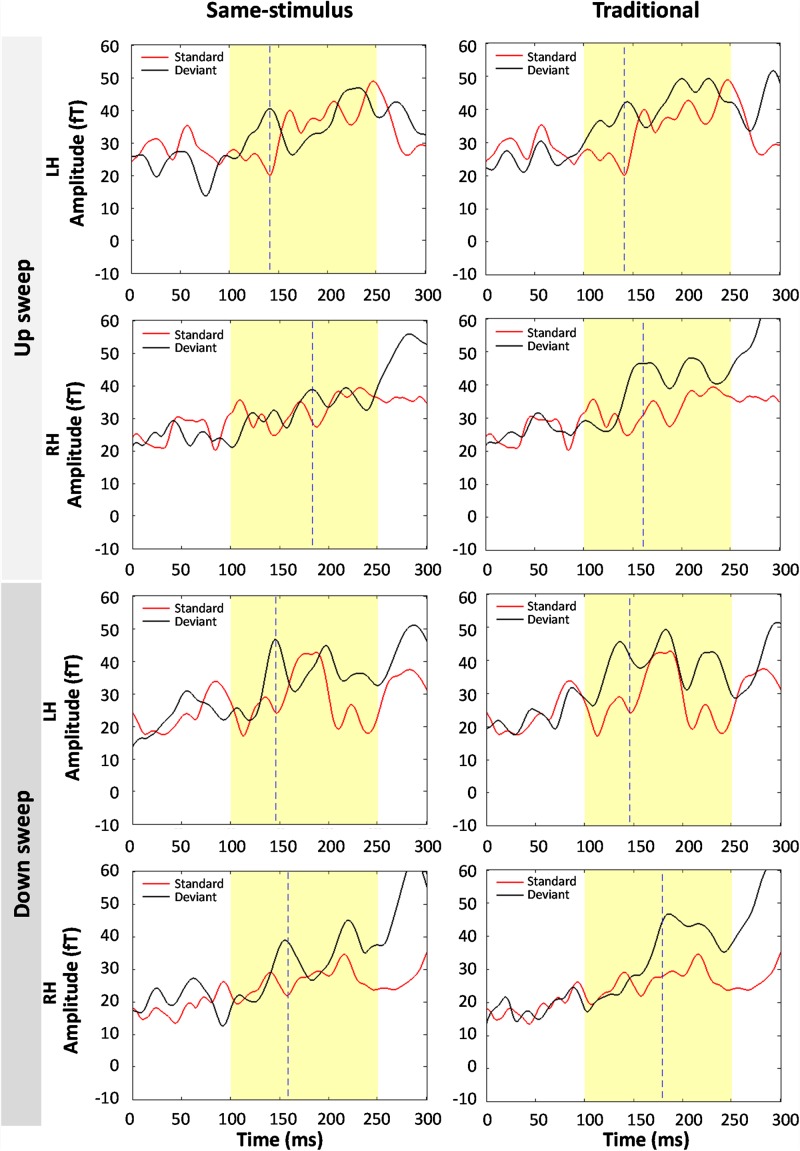
Comparison of average standard and deviant waveforms in response to 10-ms FM sweeps using same-stimulus **(left panel)** and traditional calculations. **(Top four panels)** show the magnetic field responses to upward FM sweeps and **(bottom four panels)** show downward sweeps. LH and RH indicate response in the left and right hemispheres, respectively. Dashed line indicates the peak latency of the MMNm response. No significant difference in average MMNm amplitudes was observed in both same-stimulus and traditional methods.

Figure 7 shows the average standard and deviant waveforms elicited by the shortest duration (10-ms) FM sweeps using same-stimulus and traditional methods of calculation. A significant MMNm response was observed for the 10 ms sweep condition, *t*(59) = 11.538, *p* < 0.01. A three-way repeated measures ANOVA was performed to evaluate the effect of method of calculation, hemisphere and direction on the RMS of the MMNm response elicited by the 10-ms FM sweep. There was a significant main effect of sweep direction on MMNm response at 10-ms sweep duration, *F*(1,13) = 8.29, *p* < 0.05. Down sweep elicited a larger MMNm response than up sweeps at the 10 ms duration. No hemispheric difference of MMNm response pattern was observed at the 10-ms duration, *F*(1,13) = 0.134, *p* = 0.720. The effect of method of calculation on the MMNm response elicited by the 10-ms sweep was not significant, *F*(1,13) = 0.003, *p* = 0.955. There was no significant interaction between the direction of FM sweep and method of calculation, *F*(1,13) = 3.237, *p* = 0.095. The interaction between hemisphere and sweep direction on MMNm response was not significant at this duration, *F*(1,13) = 1.633, *p* = 0.224.

### Behavioral Data

**FIGURE 8:**
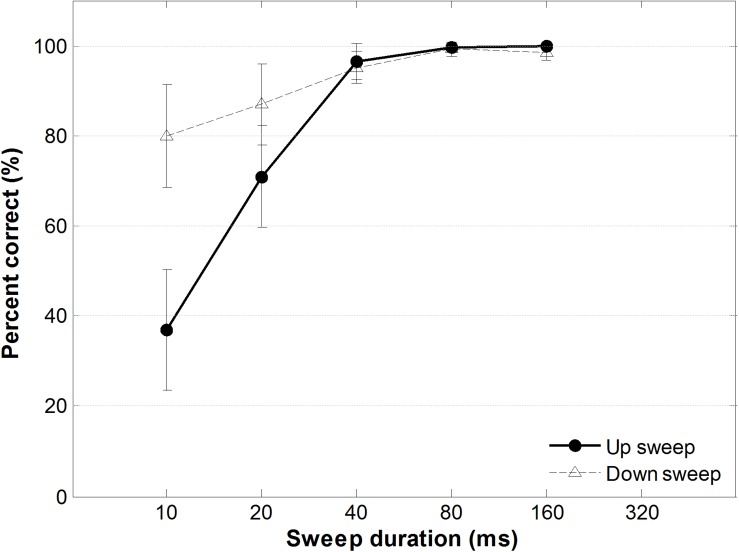
Performance accuracy as a function of sweep duration of the FM sweep behavioral identification task. Solid and dotted lines indicate accuracy for identifying upward and downward frequency sweeps, respectively. Error bars represent ±1 standard error.

Figure 8 shows percent correct performance for FM sweep direction identification task as a function of sweep duration. A two-way within-subjects ANOVA testing the effect of FM sweep direction and duration on sweep identification performance showed a significant main effect of duration, *F*(1,13) = 7.87, *p* < 0.05, a significant main effect of direction, *F*(4,52) = 88.23, *p* < 0.001, and a significant direction-by-duration interaction, *F*(4,52) = 8.70, *p* < 0.001. *Post hoc* comparisons using Bonferroni corrections (family wise α = 0.05) of different sweep durations showed that for up sweeps, identification performance accuracy between all pairs were significantly different (all *ps* < 0.001; details in Supplementary Table S1), except between the longer-duration FM sweep pairs (40 ms vs. 80 ms; 40 ms vs. 160 ms; and 80 ms vs. 160 ms). For downward frequency sweeps, however, pair wise comparison of sweep duration showed a significant difference only between the shortest duration sweep (10 ms) and the 80-ms and 160-ms FM sweeps, respectively [*t*(13) = −3.26, *p* < 0.005; *t*(13) = −3.42, *p* < 0.005]. Specifically, when the sweep duration was the shortest (fastest rate), identification performance was significantly better for downward than upward frequency sweeps, *t*(13) = −3.31, *p* = 0.006. When the sweep duration became longer than 40 ms (slower rate), identification accuracy was similar for upward and downward FM sweeps, *t*(13) = 1.623, *p* = 0.129 (for details, see Supplementary Table S1).

## Discussion

This study used high temporal resolution whole-head MEG in an MMNm paradigm to investigate the minimum temporal window required to pre-attentively detect changes in FM-sweep contour directions in the human auditory cortex. Our findings showed that listeners could implicitly, without attentional effort, differentiate the direction of non-speech signals containing linguistically relevant FM features over short durations. To our knowledge, this is the first attempt to define a temporal limit for the automatic tracking of FM-sweep directions at the cortical level. A significant MMNm response was detected, peaking in a time window 150 – 200 ms after stimulus onset. This encoding included multiple FM durations, consisting of both relatively slowly moving (i.e., 1.5 oct/s) and fast-sweeping tones (i.e., 50 oct/s) in both directions. Additionally, the MMNm response changed systematically with increasing sweep duration, with a more pronounced MMNm peak amplitude detected for upward frequency sweeps. Consistent with previous psychophysical reports, an asymmetric MMNm response pattern was observed for implicit encoding of FM sweep direction. Stronger MMNm effects were associated with increasing frequency sweep duration. In addition, the magnitude of the MMNm response elicited by FM sweeps of different durations was similar when calculated by the traditional and same-stimulus methods. This finding suggests that the upward and downward sweeps did not differ in some inherent acoustic features other than the direction of variation in pitch contour. Most importantly, we have demonstrated that listeners can implicitly differentiate FM sweep direction, even for FM sweeps at extremely high sweep rates (i.e., 50 oct/s or 10 ms). This is noteworthy given that previous psychophysical and neurophysiological studies have suggested a temporal processing threshold of 20 ms for constructing elementary auditory percepts (Poeppel, 2003; Luo et al., 2007).

### Encoding FM-Sweep Direction

We have reported more pronounced mismatch negativity responses elicited by upward sweeping tones (compared to downward sweeping tones) spanning from 10 to 320 ms when no explicit attention is required. Although downward frequency sweeps also elicited significant MMNm response across all durations, the magnitude of the response was much smaller and did not increase with sweep duration. Previous studies on the existence of asymmetric detection patterns in processing upward and downward FM sweeps have been inconsistent (Tsumura et al., 1973; Collins and Cullen, 1978; Schouten, 1985; Luo et al., 2007). The present findings suggest that Mandarin Chinese speakers pre-attentively encode frequency contours in rising FM tones better than falling FM tones, which is in line with several psychophysical and human imaging studies reporting better upward FM sweep detection pattern (Schouten, 1985; Madden and Fire, 1997; Dau et al., 2000; Gordon and Poeppel, 2002). Low-level basilar membrane structures has been offered as one plausible explanation for better detection of upward frequency sweeps, where increased synchronized neuronal firing from upward sweeps resulted in greater amplitude evoked potentials (Collins and Cullen, 1978; Dau et al., 2000; Gordon and Poeppel, 2002). Rising tonal sweeps also resulted in higher sensitivities for eighth nerve compound action potentials (Shore and Nuttall, 1985), demonstrated by cochlear microphonic potentials recorded partition displacements occurring at closer time intervals for upward sweeps (Shore and Cullen, 1984; Shore et al., 1987).

However, the opposite FM-direction sensitivity pattern has been observed in animal studies showing a higher percentage of down-preference neurons in the inferior colliculus of pallid bats (Williams and Fuzessery, 2010) and in the primary auditory cortex of cats (Mendelson and Cynader, 1985). Human behavioral studies have also reported that Mandarin Chinese speakers are better able to detect downward FM sweeps and downward pitch contours in non-speech sounds (Bent et al., 2006; Luo et al., 2007; Giuliano et al., 2011), though the existence of subject key bias may have affected the interpretation of such results, especially for binary judgment tasks. In the present study, we found that upward frequency sweeps elicited more pronounced MMNm response. To verify whether perceptual FM sweep identification performance would show a detection advantage towards upward frequency sweeps, we have also conducted a behavioral FM-sweep direction identification task with the same participants using a paradigm similar to Luo et al. (2007). The main distinctive feature of our behavioral task, unlike previous studies, was that we used a bounded FM sweep complex and thus listeners could not have used the starting frequency as a cue in determining the direction of FM sweeps. The accuracy of sweep direction identification performance was higher for downward frequency sweeps than upward sweeps, with performance reaching ceiling when sweep duration was above 40 ms (ref. Figure 8). The interaction between sweep duration and direction is interesting in that a more pronounced “detrimental” duration effect is observed for upward sweeps while performance remains approximately similar (>80%) across all durations for downward sweeps. As sweep duration decreased and the FM signal becomes more difficult to track, listeners showed a higher sensitivity to downward than upward FM sweeps. One possible interpretation is the higher frequency of occurrence for downward sweeping tones in Mandarin Chinese sharpening the perceptual sensitivity to downward frequency sweeps (Da, 2004; Zhang and Lai, 2010). The behavioral data is consistent with the sweep detection pattern reported in previous psychophysical studies (Luo et al., 2007).

### Cortical Temporal Window for FM-Sweep Direction Differentiation

Several psychophysical studies have reported a 20 ms sweep (i.e., a rate of 25 oct/s) as the minimum temporal duration required for listeners to successfully identify FM-sweep direction, suggesting it to be a fundamental integration window for the construction of auditory percepts (Schouten, 1985; Gordon and Poeppel, 2002; Luo et al., 2007). Consistent with this notion, Schouten and Pols (1989) reported that performance in FM sweep direction identification was near chance levels when the sweep duration was below the minimum temporal window (15 ms) (Schouten and Pols, 1989). In addition, the temporal window reported for sweep direction identification did not depend on whether the listeners were tone-language or non-tone language speakers (Poeppel, 2003; Luo et al., 2007). In the present study, a robust MMNm response was observed for sweeps moving as rapidly as 50 oct/s (i.e., 10 ms), suggesting the brain can implicitly differentiate the frequency direction of sweeps over extremely short durations. In our pilot study, we have also tested FM sweeps at duration of 5 ms, but could not obtain a robust MMNm response. It is worth noting that our cortical temporal window limit of 10 ms for detecting a change in FM-sweep direction is based on implicit differentiation, as indicated by a significant MMNm activity in response to a frequency direction change of the 10-ms sweep. This differs from previous psychophysical studies which required explicit overt identification of FM direction. Psychophysical studies suggests that Chinese listeners performed better in FM-sweep identification tasks because they use explicit FM direction labeling; while performance was similar to non-tone English speakers in FM sweep discrimination tasks where no explicit FM directional naming was used (Luo et al., 2007). Although we have taken steps to add steady-state tones to the sweeps, to ensure frequency direction was the only difference between the standard and deviant sounds, the potential use of other acoustic features in implicitly differentiating upward and downward sweeps remains a possibility. Regardless, the reported finding that Mandarin Chinese listeners are sensitive to fine-grained acoustic features in FM contours provides cortical evidence to the significance of frequency contour sensitivity for speech processing. This is especially critical for tone languages which require careful monitoring of changes in frequency direction.

### Deviance Direction Associated With Sound Features on Mismatch Negativity

Asymmetrical mismatch negativity response has been reported previously by increment and decrement deviance associated with various sound features such as frequency, intensity and duration. Several studies used discrete pure tones in an oddball sequence have reported increments in frequency produced higher MMN amplitudes compared to frequency decrements, lending support to an evolutionary preference for objects moving toward the listener thus sounding upward in frequency (Jacobsen and Schröger, 2001; Carral et al., 2005; Peter et al., 2010; Karanasiou et al., 2011; Ruusuvirta and Astikainen, 2012). Our finding of a perceptual preference for downward sweeps contradicts the augmented MMN effect reported for frequency increments compared to frequency decrements. One possible explanation is that the frequency of occurrence for downward sweeping tones (i.e, Tone 4; 42.5%) is much higher compared to rising tones (i.e., Tone 2; 18.4%) in Mandarin Chinese, thus contributing to the enhanced sensitivity observed for down FM sweeps in Mandarin Chinese speakers (Da, 2004; Zhang and Lai, 2010). Another interpretation could be the dissociation between behavioral data and the mismatch negativity response elicited by the same FM sweep stimulus. In fact, for the electrophysiological data, we observed a larger MMNm amplitude associated with rising FM sweeps compared to falling FM sweeps, which corroborates previous evidence that increments in frequency produced higher MMN amplitudes compared to frequency decrements. Such a discrepancy between behavioral data and electrophysiological response in the processing of deviance direction of acoustic features has also been reported in previous studies (Grimm et al., 2004; Colin et al., 2009; Karanasiou et al., 2011; Shestopalova et al., 2018). This suggests that our finding of similar MMNm amplitudes elicited by the down sweeps at different duration is not related to a deficit in the discrimination process (as perceptual accuracy in the same condition was above 80% for downward FM sweep), but may be exclusively cortical phenomenon. However, we did take caution that here we utilized a stimulus with a continuous frequency increment which differed from previous frequency increment/decrement MMN studies that primary used discrete tones with a fixed frequency difference. Future studies can further compare the increment and decrement frequency MMN elicited by frequency change formed by discrete and continuous tones with the same magnitude of pitch interval.

In terms of sound intensity, some studies have reported an asymmetry in the mismatch negativity and psychophysical detection for rising level sounds compared to falling level sounds (Jacobsen et al., 2003; Shestopalova et al., 2018) while others have reported no differences (Altmann et al., 2013). Interestingly, the reported priority for the processing of rising intensity sounds has been suggested to be associated with an intrinsic pre-attentional processing possibly related to stimulus detection (Bach et al., 2008). One may wonder whether the perceived loudness of the FM sweeps is a factor that contributes to the perceptual preference for downward frequency sweeps observed in the present study. The sweep frequency range is between 600 and 900 Hz in this study, which falls on a relatively flat line on the standard equal loudness curve. In addition, we have taken measures to ensure that the perceived loudness between upward and downward sweeps are equivalent by attenuating the boundary tones. Thus we speculate that the asymmetry in perceptual response observed for upward and downward sweeps is not likely due to perceived loudness. The similar MMNm results obtained using same-stimulus and traditional calculation methods supports the notion that the response asymmetry is more likely due to pitch contour change, rather than inherent stimulus differences such as loudness. However, future studies can consider using a more refined individualized equal loudness curve measured over the entire sweep frequency range for different durations. Furthermore, studies investigating the mismatch negativity evoked by sound duration contrasts have revealed an asymmetry of MMN amplitude for duration increment and decrement (Jacobsen and Schröger, 2003; Okazaki et al., 2006; Colin et al., 2009) which could be further modulated by the spectral complexity of the stimulus (Takegata et al., 2008). Specifically, shorter-duration deviants elicited larger MMN amplitude than for longer-duration deviants (Colin et al., 2009). Importantly, most studies indicate an increase in MMN amplitude corresponding to an increase in duration difference. Our findings of an increase in MMNm amplitude corresponding to different magnitude of FM sweep duration is consistent with studies reporting different magnitude of duration change represented in mismatch negativity response (Kaukoranta et al., 1989; Joutsiniemi et al., 1998; Okazaki et al., 2006).

### Issues Concerning Potential Habituation Effect on MMN Response

One potential concern is whether the MMN response amplitude can be affected by neural habituation due to repetitive presentation of the same stimulus over time. One measure we have taken to minimize the risk of habituation effect is to reduce each sequence length to less than 10 min and minimize the number of deviant stimulus presented per sequence (McGee et al., 2001). Another concern regarding potential neural habituation is whether there is a difference in the habituation effect on the MMNm response due to different FM-sweep stimulus durations. In this study, we used a constant inter-stimulus interval (ISI) for each sequence whereas the FM sweep stimulus duration is the only parameter that varies across blocks (i.e., different SOA). If different FM-sweep duration leads to different degree of neural habituation, then the amplitude of the MMN response may be differentially affected. Previous studies investigating the habituation effect on the auditory ERP in relation to stimulus duration have reported no effect of tone duration (50, 100, 200 ms) on the degree of neural habituation (Rosburg et al., 2002). In addition, several studies have reported that MMN amplitude decreased when the SOA increased (Mäntysalo and Näätänen, 1987; Pegado et al., 2010). Accordingly, one would expect that the longest duration FM sweep block (i.e., longest SOA) should evoke the smallest MMN response. In fact, we observe the highest MMNm amplitude corresponding to the longest duration FM sweeps condition and the MMNm amplitude progressively declines with decreasing FM sweep duration. Taken together, the use of short stimulus sequence and evidence from previous studies suggest that the neural habituation due to different FM stimulus duration may have insubstantial effect on the MMNm response.

### FM Sweep Processing and Speech

These findings contribute to existing psychophysical evidence for FM-sweep processing by suggesting that tone-language speakers are implicitly aware of the fast-changing frequency variations in FM sweeps at the cortical level. We were able to observe for the first time a pronounced MMNm elicited by an extremely short sweep duration of 10 ms, a temporal window that is much shorter than previously suggested. The perceptual accuracy in identifying downward sweeps especially at short sweep durations also corroborates the finding of a 10 ms temporal integration window for constructing an elementary auditory percept. Specifically, at the shortest duration (10 ms) sweep when the FM stimulus is sweeping quite fast and difficult to track, identification performance is still around 80% for downward sweeps. Although the perceptual sensitivity to processing rising frequency sweeps is much lower at the 10 ms duration, a pronounced MMNm response is still evoked at the cortical level. The proficiency and speed with which the auditory cortex is able to automatically analyze FM sweep contours supports the important relationship between FM-sweep processing and speech encoding. The importance of FM processing for Mandarin Chinese listeners in order to decode word meaning has been proposed to contribute to their enhanced ability to perceptually analyze FM tones (Luo et al., 2007). Our finding is in line with some research evidence suggesting that the auditory cortex sensitivity to FM sweep is related to speech processing. For instance, Joanisse and Gati (2003) showed that consonants and FM tones yielded stronger activation in the left auditory regions, whereas pure tones with the same FM features did not. Similar evidence for a link between FM sweep and speech encoding includes the finding that dyslexic individuals exhibit a reduced neural MMNm response to FM signals (Stoodley et al., 2006), and that deficits in FM-sweep processing is associated with reading skills and developmental dyslexia (Witton et al., 1998). Others have reported that phonologically impaired patients have difficulty processing FM signals (Talcott et al., 2002).

Some research have suggested that tone language speakers, and in some cases, musicians, show enhanced pitch processing ability in other nonlinguistic domains such as music (Luo et al., 2007; Pfordresher and Brown, 2009; Bidelman et al., 2011; Giuliano et al., 2011). One speculation is that tone languages (compared to non-tone languages) use pitch height and pitch changes to convey lexical information, and that proficiency in a tone language requires forming strong associations between pitch changes and word meanings (Yip, 2002). As such, the acquisition of a tone language has been hypothesized to sharpen the processing of other pitch-relevant auditory features in speech and or other non-speech domains. For instance, tone-language speakers have been shown to discriminate and imitate through singing musical pitch better than non-tone language speakers both at the perceptual level (Luo et al., 2007; Pfordresher and Brown, 2009; Giuliano et al., 2011) and at the brainstem level (Bidelman et al., 2011). Along the same line, Xu et al. (2006) demonstrated that Mandarin Chinese (i.e., tone language) speakers showed similar categorical perception of the pitch contour in speech stimuli and their non-speech homologous harmonic tone, whereas English (i.e, non-tone language) speakers did not. These findings suggest that the acquisition of a tone language may possibly fine-tune the processing of non-speech auditory features such as musical pitch or frequency modulation (Xu et al., 2006; Pfordresher and Brown, 2009). The present study provides cortical evidence of a temporal window threshold within which tone language speakers were able to encode extremely fast changes in frequency sweep contours. Although our finding is in line with the aforementioned evidence supporting the relationship between tone language acquisition and frequency sweep encoding; the specific acoustic features that is modulated by tone-language experience requires further investigation.

## Conclusion

We report a very short temporal window limit of 10 ms for the automatic tracking of a dynamic change in frequency sweep direction at the pre-attentive cortical level. The cortically evoked MMNm response is modulated systematically by sweep duration with more pronounced temporally related enhancement for rising frequency-sweep contours. Falling frequency-sweep contours elicits similar extent of mismatch negativity responses across all sweep durations. That the cortically evoked mismatch negativity response is extremely temporally sensitive and systematically affected by FM sweep direction and rate provides further evidence for the special status of FM sweep contour in speech encoding.

## Data Availability Statement

The datasets generated for this study are available on request to the corresponding author.

## Ethics Statement

This study was carried out in accordance with the recommendations of the Research Ethics Committee of National Taiwan University with written informed consent from all participants. All participants gave written informed consent in accordance with the Declaration of Helsinki. The protocol was approved by the Research Ethics Committee of National Taiwan University, Taiwan.

## Author Contributions

S-JK, DW, and I-HH designed the experiment. S-JK and I-HH programmed the auditory stimuli and experimental tasks. C-HH contributed to MEG data analysis. All authors contributed to data collection. I-HH, S-JK, and DW prepared the figures and interpreted the results. I-HH wrote the manuscript.

## Conflict of Interest

The authors declare that the research was conducted in the absence of any commercial or financial relationships that could be construed as a potential conflict of interest.
