# Supplementary material for: A Minimum Temporal Window for Direction Detection of Frequency-Modulated Sweeps: A Magnetoencephalography Study
Source: Front Psychol. 2020 Mar 10;11:389. doi: 10.3389/fpsyg.2020.00389 (PMC7078663; doi:10.3389/fpsyg.2020.00389)
Supplement: Supplementary file 1 [file Table_1.DOCX]

**Supplementary File**

**Table S1 Detailed post-hoc statistics for behavioral data**

**Up Sweep**

|  | Bonferroni-adjusted statistics | | |
| --- | --- | --- | --- |
| Duration (ms) | *t* | *df* | *p-value 2-tailed*  *(based on*$\alpha$*/10)* |
| 10 vs. 20 | -5.611 | 13 | <0.001* |
| 10 vs. 40 | -7.077 | 13 | <0.001* |
| 10 vs. 80 | -8.616 | 13 | <0.001* |
| 10 vs. 160 | -8.521 | 13 | <0.001* |
| 20 vs. 40 | -3.698 | 13 | 0.003* |
| 20 vs. 80 | -4.558 | 13 | 0.001* |
| 20 vs. 160 | -4.522 | 13 | 0.001* |
| 40 vs. 80 | -1.146 | 13 | 0.273 |
| 40 vs. 160 | -1.461 | 13 | 0.168 |
| 80 vs. 160 | -1.000 | 13 | 0.336 |

**Down Sweep**

|  | Bonferroni-adjusted statistics | | |
| --- | --- | --- | --- |
| Duration (ms) | *t* | *df* | *p-value 2-tailed*  *(based on*$\alpha$*/10)* |
| 10 vs. 20 | -1.718 | 13 | 0.109 |
| 10 vs. 40 | -3.073 | 13 | 0.009* |
| 10 vs. 80 | -3.256 | 13 | 0.006* |
| 10 vs. 160 | -3.415 | 13 | 0.005* |
| 20 vs. 40 | -1.806 | 13 | 0.094 |
| 20 vs. 80 | -2.542 | 13 | 0.025 |
| 20 vs. 160 | -2.589 | 13 | 0.022 |
| 40 vs. 80 | -1.918 | 13 | 0.077 |
| 40 vs. 160 | -2.510 | 13 | 0.026 |
| 80 vs. 160 | .634 | 13 | 0.537 |

**Up vs. Down**

|  | Bonferroni-adjusted statistics | | |
| --- | --- | --- | --- |
| Duration (ms) | *t* | *df* | *p-value 2-tailed*  *(based on*$\alpha$*/3)* |
| 10 | -3.312 | 13 | 0.006* |
| 20 | -1.697 | 13 | 0.113 |
| 40 | 1.623 | 13 | 0.129 |
